# Poster Session II - A287 DIAGNOSTIC DILEMMA: INTESTINAL TUBERCULOSIS MIMICKING TERMINAL ILEAL CROHN’S DISEASE

**DOI:** 10.1093/jcag/gwaf042.287

**Published:** 2026-02-13

**Authors:** R Kakkar, J Guo

**Affiliations:** Division of Gastroenterology, Department of Medicine, University of British Columbia, Vancouver, BC, Canada; Division of Gastroenterology, Department of Medicine, University of British Columbia, Vancouver, BC, Canada

## Abstract

**Background:**

Intestinal tuberculosis (TB) accounts for 1-3% of TB cases worldwide and risk factors include malignancies (especially lymphoma), use of corticosteroids, and anti-TNF agents. Presentations are non-specific, including abdominal pain, fever, weight loss, altered bowel habits, and bleeding. Complications include obstruction and, less commonly, perforation which can occur even during treatment. Diagnosis is challenging as clinical and endoscopic features overlap with Crohn’s disease. Moreover, acid-fast bacilli (AFB) are detected in only 20% of biopsies and PCR shows 65% sensitivity for TB. Given these diagnostic limitations, a high index of suspicion is essential for timely diagnosis and management.

**Aims:**

To describe a presentation of intestinal TB complicated by small bowel obstruction and perforation in the context of glucocorticoid use, highlight the diagnostic challenges given overlapping features with Crohn’s disease, and emphasize the role of pre-treatment TB screening.

**Methods:**

A detailed case review was conducted of a patient with intestinal TB initially diagnosed with Crohn’s disease and developed intestinal perforation while on corticosteroids. Clinical course, laboratory data, imaging, endoscopy, and histopathology were analyzed.

**Results:**

Mr. A is a 36-year-old male from Ghana who presented with several months of progressive abdominal discomfort. Imaging showed a high-grade small bowel obstruction with a terminal ileal stricture. Initial biopsies demonstrated mild architectural distortion and a poorly formed non-necrotizing granuloma. AFB stains were negative. Based on these findings, a diagnosis of Crohn’s disease was made, and he was started on corticosteroids and discharged on a taper. Pre-treatment screening later returned positive for both tuberculin skin test and interferon-gamma release assays.

Repeat colonoscopy showed persistent ileocecal stricture with unremarkable mapping biopsies. While on corticosteroids, he re-presented with severe abdominal pain and was found to have localized small bowel perforation requiring surgical resection. Given his epidemiologic risk factors, positive TB screening, and compatible presentation, he was diagnosed with intestinal tuberculosis and started on anti-tuberculous therapy with clinical improvement.

**Conclusions:**

This case highlights the difficulty in distinguishing intestinal TB from Crohn’s disease due to overlapping clinical, endoscopic, and histologic features along with low yield of AFB staining and PCR. Given the distinct management and risks of misdiagnosis, clinicians should maintain a high index of suspicion for TB in patients with risk factors. Early screening and interpretation of results are essential to ensure timely and appropriate treatment.

**Funding Agencies:**

None

